# Questions and Consequences of Omics in Genetically Engineered Crop Regulation

**DOI:** 10.1002/pei3.70033

**Published:** 2025-02-17

**Authors:** Asa Budnick, Eric Butoto, Nick Loschin, Amanda Mainello‐Land, Jill Furgurson, Rebekah Brown, Greg Ferraro, Rex Alirigia, Modesta Abugu, Ruthie Stokes, Christopher Gillespie, Nolan Speicher

**Affiliations:** ^1^ Plant and Microbial Biology North Carolina State University Raleigh North Carolina USA; ^2^ Genetic Engineering and Society Center North Carolina State University Raleigh North Carolina USA; ^3^ Crop and Soil Sciences North Carolina State University Raleigh North Carolina USA; ^4^ Applied Ecology North Carolina State University Raleigh North Carolina USA; ^5^ Entomology and Plant Pathology North Carolina State University Raleigh North Carolina USA; ^6^ Forestry and Environmental Resources North Carolina State University Raleigh North Carolina USA; ^7^ Food, Bioprocessing and Nutrition Sciences North Carolina State University Raleigh North Carolina USA; ^8^ Agricultural and Resource Economics North Carolina State University Raleigh North Carolina USA; ^9^ Horticultural Science North Carolina State University Raleigh North Carolina USA; ^10^ Biochemistry North Carolina State University Raleigh North Carolina USA; ^11^ Communication, Rhetoric, and Digital Media North Carolina State University Raleigh North Carolina USA

## Abstract

In 2016, a National Academies of Sciences, Engineering, and Medicine advisory committee proposed omics technologies as one possible adequate response to the regulatory challenges posed by gene editing and synthetic biology. This paper presents a set of questions that would need to be answered to integrate omics experiments and data into crop regulatory systems. These questions concern both experimental practice and how omics‐experimental and regulatory systems intersect. We anticipate that the chosen answers to these questions will impact the scientific validity, regulatory burden, and usefulness for forecasting risk in nuanced ways. In doing so, we conclude that the integration of omics technologies into regulatory systems poses an array of more‐than‐technical dilemmas whose management will require cross‐sector collaboration and innovative approaches to socio‐technical decision‐making.

## Graphical abstract

Omics technologies have been proposed as a solution to some issues in genetically engineered crop regulation. We took an interdisciplinary approach to reviewing unanswered questions in one of these proposals and considering some potential consequences across areas of concern.

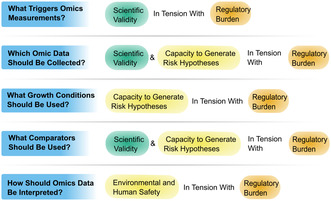

## 1 Introduction

The term “omics” refers to a suite of techniques that aim to precisely quantify biomolecules in cells, tissues, and organisms. Researchers commonly use omics to analyze genomic, transcriptomic, proteomic, or metabolomic data, all requiring different sets of instruments and procedures. Because of innovation in the biotechnology sector, omics technologies are increasingly accessible and robust, namely in their ability to assist breeders in developing novel agricultural traits (Wang et al. 2019). In addition to their use in commercial agribusiness, the maturation of omics technologies has coincided with calls for their use in regulatory settings. These date back to at least 2016, when a report from the National Academies of Sciences, Engineering, and Medicine (NASEM 2016, 3) proposed “a tiered approach to regulation that is based in part on new‐omics technologies that will be able to compare the molecular profiles of a new variety and a counterpart already in widespread use.” In this tiered approach, a distinction is made between formalized risk and safety assessments conducted by regulatory agencies and pre‐assessment screening activities that would occur upstream of those evaluations. The NASEM report does not recommend the use of omics in safety testing, but instead discusses its potential in pre‐assessment screening, which would help determine which new varieties should undergo more robust and formalized risk and safety evaluations and which should not (NASEM 2016).

As laid out by the committee, the hypothetical use of omics in pre‐assessment screening would involve comparative analyses between a new variety and those already in use. If this analysis revealed no differences (category 1) or differences with “no expected health or environmental effects” (category 2), then the new variety could enter the commercial market with no formal safety evaluation. Alternatively, if differences were observed with “potential for health or environmental effects” (category 3) or if the differences were unable to be interpreted (category 4), then the new variety would undergo a more formal risk and safety assessment through the appropriate regulatory agencies (see figure 1 in Gould et al. 2022, 1052). Table 1 summarizes these categories.

**TABLE 1:** Summary of proposed omics pre‐screening categories.

Category	Category definition	Outcome
Category 1	No differences between the varieties	No further testing
Category 2	Understood differences with no expected outcome on health and environmental effects	
Category 3	Understood differences with potential for health or environmental effects	Further testing
Category 4	Differences that cannot be interpreted	

In sum, the proposal offered by the NASEM committee rests on the ability of omics technologies to inform two critical questions: (1) Is the molecular profile of a new variety different? And (2) is the difference meaningful? Answering the first question differentiates Category 1 varieties from all others; answering the second question differentiates between category 2 and categories 3–4. Although we share the committee's view that such a tiered approach could help move crop governance in the direction of a more product‐based regulatory environment *in theory*, we also anticipate significant questions about how such a system would be implemented in practice. The goal of this paper was to examine those questions and hypothetical chosen answers more closely. More specifically, we highlight a series of key questions and consequences that we expect could accompany the tiered approach summarized above. These questions are pivotal not only because they affect broader regulatory concerns but also because they are immensely complex because they cannot be answered by science alone.

The general outline of the paper is as follows. First, we briefly review ongoing discussions about the general use of omics in regulatory environments, which has become a polarizing issue in the scientific community. We then introduce three regulatory concerns that characterize these debates and thus are bound to be impacted by choices made in the practical implementation of omics in pre‐assessment screening. From there, we discuss a series of questions that we expect to be generally applicable and important in consideration of the NASEM committee's tiered approach. Taking a holistic view of these decision points and their intersections with broader concerns, we conclude in suggesting that these decisions are as much a question of value‐laden judgment as they are observable facts and thus will require innovative practices in sociotechnical decision‐making. Although we do not propose any particular practice here, we point to existing literature in science and technology studies that can offer insight into managing the complexities of these decisions.

### 1.1 Omics in Agricultural Biotechnology Regulation

Using omics to inform decision‐making in the regulatory oversight of agricultural products is a contentious idea and not technically straightforward, as omics technologies are still rapidly evolving (Dai and Shen 2022). Some experts have argued that incorporating omics is impractical and unnecessary and would not improve the risk assessment of GE products (Delaney et al. 2019; Fedorova and Herman 2020). Scientists have argued that growth conditions and genetic backgrounds will cause more variance in omics measurements than those attributed to GE modification (Lewis et al. 2008; WHO and FAO 2009). Another criticism of this approach is that additional testing would be costly and would not necessarily help improve consumer acceptance (Delaney et al. 2019). Lastly, omics methods have been criticized as non‐hypothesis‐driven because they are not targeted to specific molecules with known risks, and most plant molecules do not have a significant impact on the safety of foods (Bedair and Glenn 2020).

On the other hand, targeted compositional analyses can be critiqued as biased or limited in ways that reflect the monetary interests of developers (Millstone et al. 1999). With this in mind, some researchers have suggested that the unbiased nature of omics is key to its success (Gong and Wang 2013). Proponents of incorporating untargeted omics techniques into the regulatory process argue that the data can inform risk hypotheses that would not otherwise have been tested with targeted analysis (Benevenuto et al. 2023; Chu and Agapito‐Tenfen 2022). A meta‐analysis conducted by Chu and Agapito‐Tenfen (2022) found various unintended changes to the genome across several plant species and GE techniques. They observed that large genetic changes were best detected with untargeted methods such as whole genome sequencing (WGS). Benevenuto et al. (2023) made metabolomic and proteomic measurements on GE and non‐GE soybean lines and found 43 proteins differentially expressed in the GE line, which showed similarity to known allergens. Because the proteins would not have been measured in a typical targeted analysis, the findings suggest that untargeted omics technologies can reveal unintended changes that may be relevant to generating risk hypotheses. The presence of unintended changes does not mean that a crop necessarily poses a new or increased risk (NASEM 2016); however, characterizing these unintended changes in both GE and non‐GE crops may inform testable risk hypotheses. In cases where omics reveals no statistically significant and/or risk‐relevant changes, it can provide evidence that further testing is unnecessary.

## 2 Questions and Concerns for Including Omics in Regulation

**FIGURE 1:**
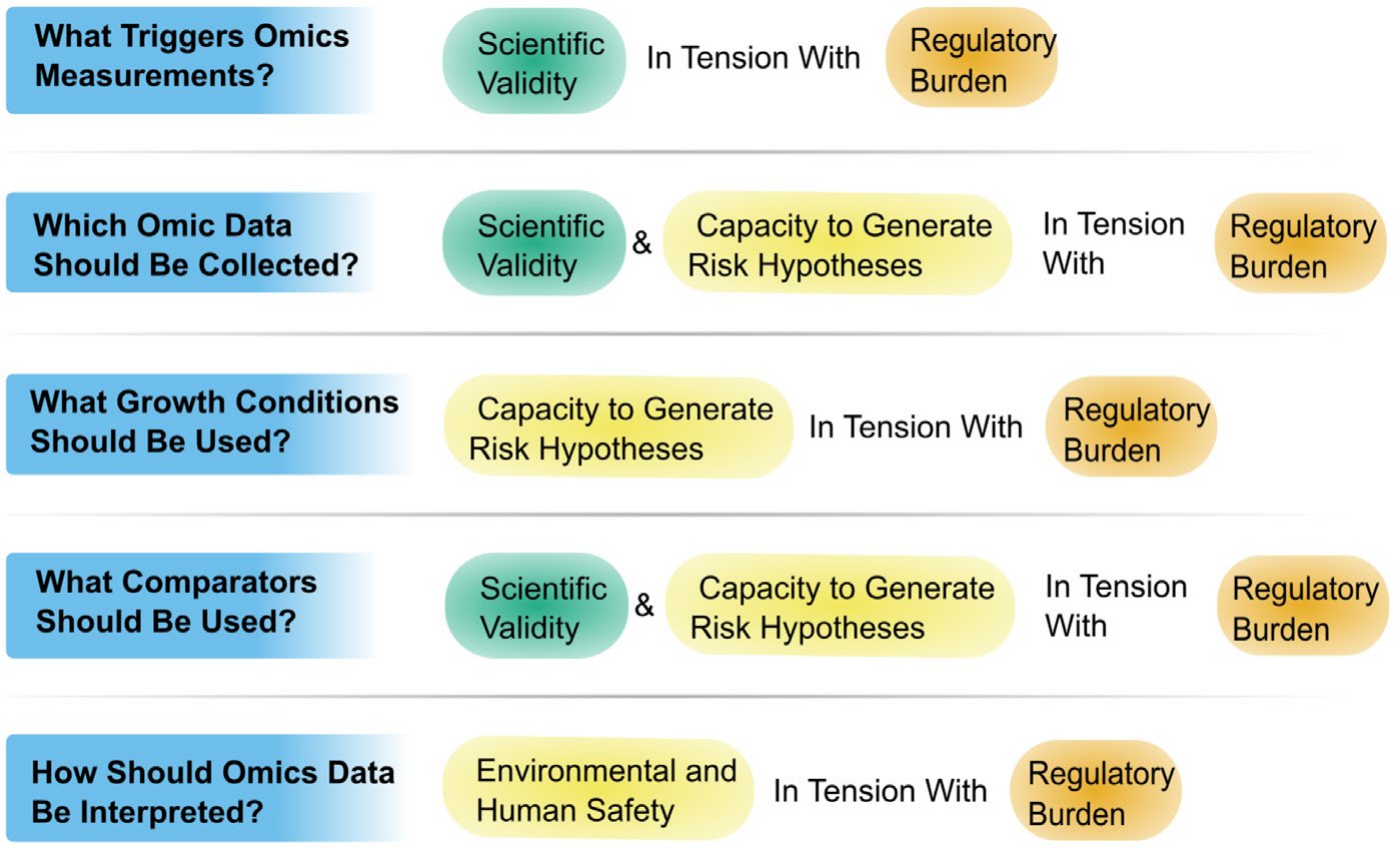
Visual summary. Questions posed here are summarized alongside a simplification of the areas of regulatory concern that we foresee them impacting. This simplified view is not meant to portray the nuances in how specific choices may differently impact areas of regulatory concern; additionally, more tensions may exist and only main tensions are shown.

Changes to the regulatory system are likely to have diverse impacts and affect various concerns. In this paper, we focus on three regulatory concerns—scientific justification, regulatory burden, and capacity to inform risk hypotheses, which we anticipate being impacted by choices in a hypothetical crop regulatory system that incorporates omics. In the context of this paper, scientific justification refers to the alignment of the regulatory system with current scientific understanding. Regulatory burden refers to perceived and actual costs the regulatory system poses to developers and regulators, and capacity to inform risk hypotheses refers to the ability to utilize omics data to meaningfully direct follow‐up assessment. We chose these particular concerns because they carry some similarity to previous work that has offered a set of criteria for evaluating biotechnology oversight, and we expect the choices made in incorporating omics into a regulatory system to offer trade‐offs between these regulatory concerns (Gordon et al. 2021; Kuzma 2023) (Figure 1).

### 2.1 What Triggers Omics Analysis?

The NASEM committee's tiered approach suggests the use of omics in pre‐assessment screening for any new crop variety intended for commercialization, regardless of what technique was used to create it (Gould et al. 2022; NASEM 2016). This, in essence, means that intending to release any new variety is the trigger for omics pre‐assessment. Evaluating specific triggers is outside of the scope of this paper, but we hope to illustrate that the choice of a trigger for omics screening puts the scientific validity of the regulatory system in tension with the regulatory burden of releasing new crop varieties.

What does the NASEM committee's trigger of ‘any new variety’ mean in practice? One useful proxy for estimating the number of both GE and non‐GE new crop varieties that are produced every year is the USDA's Plant Variety Protection (PVP) certificate that is administered by the agency's Plant Variety Protection Office (PVPO). According to a 2023 report, the PVPO receives “approximately 500 applications per year” (USDA 2023). Assuming that an application for a PVP is synonymous with a new variety, one way omics could be implemented into pre‐assessment screening is to require that it be conducted on all PVP applications. This would be a significant departure from existing regulations and impose a much higher regulatory burden as most varieties submitted to PVP are conventional crops that currently have few premarket regulatory costs. However, there may be alternative ways to envision the incorporation of omics into pre‐assessment screening of both GE and non‐GE varieties. Triggers for omics, which are less broad, may be more appropriate in terms of trade‐off between scientific justification and regulatory burden. For example, triggers for omics analyses could be based on the anticipated scale of production of each variety and differ for different types of crops. Imagine a regulatory trigger is set at a certain acreage, and product crops grown above this acreage threshold would be subject to omics screening. This assumes that varieties that are grown on fewer acres are less likely to pose significant risk, which cannot be the case for all crops and all risks. For example, some crops that are eaten directly by humans and grown on relatively few acres may have a greater capacity for human safety risks than commodity crops fed to livestock. A trigger for omics screening that applies equally to GE and conventional new varieties would be considered scientifically justified by some; however, the broader the trigger, the higher the regulatory burden. Attenuating this trade‐off to focus triggers on the basis of where new varieties may be subjected to omics screening in a risk‐proportionate manner is an important first decision that would need to be made.

### 2.2 What Omic Data Should Be Collected?

Choosing which omics measurements are necessary for a regulatory system is an important decision because the type and degree of omics data collected influence both costs and utility (Table 2). Regulatory bodies would need to set the type and degree of omics requirements on the basis of various factors. For example, the European Food Safety Authority has set standards on the depth of sequencing for WGS because the utility of WGS data depends on having sufficient sequencing depth and coverage (European Food Safety Authority [EFSA] et al. 2024). Deciding which types of omics data are collected, and for which specific uses, will have an impact on the regulatory burden (i.e., cost) and the ability to inform risk hypotheses for further testing. Furthermore, crops are different in ways that affect the cost and complexity of gathering and interpreting omics data—for example, maize has a larger genome than soybean. Because of this, standards for omics data collection may need to be set specifically for each crop species. Currently, there are significant differences in costs across omics methods, but as the technical capacity to do omics improves, it is likely that this will be ameliorated over time (Jaramillo‐Botero et al. 2022).

**TABLE 2:** Examples of the various types of omics approaches that may be relevant to crop regulation. Other omics methods exist, but these are the most common and pertinent to this discussion.

Ome	Description	Utility	Limitation
Genome	The complete set of DNA with an organism or cell.	Useful for monitoring and categorizing DNA sequences. Some DNA changes have predictable consequences that could inform risk hypotheses with appropriate biological knowledge.	In many cases, DNA sequence changes do not provide enough information to predict risks.
Transcriptome	The complete set of RNA transcripts (or gene expression) in a tissue.	RNA expression and degradation of known or unknown genes can be evaluated and differences between plants can be measured.	RNA expression changes rapidly and is variable making it difficult to collect and interpret. It is not always an accurate proxy for protein abundance depending on collection, extraction, and sequencing.
Proteome	The complete set of protein expression in a cell.	More stable and important for risk assessment as some plant proteins represent toxins, antinutrients, and allergens.	Identification and characterization of proteins is more challenging and expensive than characterizing genomes and transcriptomes.
Metabolome	The complete set of molecules (metabolites) in a sample.	Useful for understanding underlying biochemical activity and state of cells.	The costs associated with these measurements are high. No metabolomic method has been established in plants that can measure and identify all classes of plant metabolites without bias.

### 2.3 Which Growth Conditions Should Be Used?

Plants can be grown in controlled conditions (such as in a greenhouse where light, water, and temperature are controlled) or in field conditions. Field conditions can vary widely, and some omics experiments involving crops have found greater differences caused by environmental factors rather than by genotypic differences (Barros et al. 2010; Frank et al. 2012). Gould et al. (2022) suggest using omics to compare a new variety with existing varieties grown across various environments. This experimental setup allows for environmental variance to be estimated and differences attributed to the varieties' genetics. Controlled growth conditions may be cheaper and limit environmental variance. However, omics profiles generated from field trials may be more analogous to production crops and be more beneficial for risk hypothesis generation. Field experiments are also technically difficult, meaning that the regulatory burden associated with them comes not only in cost but also in the level of expertise required to manage complex experiments and analyses across many field sites.

Growth conditions can also shape plant responses in ways that may be meaningful for regulatory approval. Plants have a variety of mechanisms to respond to stressors; some of these stress response pathways are relevant to risk assessment because they can lead to the expression of plant toxins and other characteristics that may only become apparent under specific and relevant growth conditions (Benevenuto et al. 2022; Gullì et al. 2015; Lannoo and Van Damme 2014). For example, potatoes express glycoalkaloid toxins differently depending on diverse stresses including water, light, and temperature (Love et al. 1994). Expanding the number of growth conditions in omics experiments not only improves the capacity for generating risk hypotheses but also increases the regulatory burden by increasing the cost of omics experiments and the expertise required to manage and analyze complex experiments. Plant growth conditions are therefore a key choice in a hypothetical omics regulatory system that may be viewed differently by developers, consumers, and regulators.

### 2.4 What Comparators Should Be Used?

At the core of the NASEM committee's proposal to use omics in pre‐assessment screening is comparative analysis between experimental samples and existing varieties. In the plant omics experiments relevant to this discussion, the new product plant is contrasted with other varieties, which are comparators. However, exact standards for comparators have not been determined. Comparators could be from the same experiment or drawn from an existing database. The number and type of comparators directly impact the results of omics experiments. Comparator plants can include similar varieties or near‐isogenic plants, which are genetically identical to a GE plant except for lacking the GE component. A wider set of comparators is expected to produce a broader range of omics measurements, which makes it less likely that a new variety's omics profiles would be outside of the range of the comparators'. A regulatory system that incorporates omics data will need to decide what comparators should be used in omics experiments because experimental conclusions are influenced by the inclusion or exclusion of specific comparators.

Standards on which comparators are used need to be set as they directly influence the results of the analyses. For example, when Monsanto (now Bayer Crop Science) shared data with the European Food Safety Authority (EFSA) comparing GE and non‐GE soybean varieties, significant differences between the varieties were clear when a narrow set of reference data was used to compare. However, these differences were insignificant when a larger dataset of published results was added in and used to compare the varieties (Benevenuto et al. 2023). The choice of comparators impacts the scientific validity of the regulatory system and the moderation of developer incentives.

Omics data from comparators can either be from the same experiment or from previous experimental data. In the latter case, a relevant database is key for benchmarking and drawing reliable conclusions (Brooks et al. 2024). Appropriate comparators must have matching treatments such as environment, light, temperature, and pathogen pressure for an accurate comparison without confounding variables (Kok et al. 2019; Schwanz et al. 2022; Van Dijk et al. 2014). Using comparator plants grown in the same experiment limits variance from confounding variables that are accounted for in databases. This increases the interpretability of an omics analysis and may attribute more differences to the genotype of the new variety, GE or otherwise. This could offer gains in generating safety and environmental risk hypotheses as the resulting comparisons would show narrower and more interpretable differences. This comes at a cost, however, as growing comparators in the same omics experiment increases the size and cost of the experiment and would increase the regulatory burden. The choice to allow comparators drawn from databases may differentially burden different types of crops. For well‐studied commodity crops such as maize, soybean, and cotton, there is a larger body of existing omics data that could be leveraged to reduce the regulatory burden of new omics analyses (Gui et al. 2020; Liu et al. 2023; Yang et al. 2023). Crops without prior bodies of omics research would need to include comparators within each experiment, making those experiments more expensive.

### 2.5 How Should Omics Data Be Interpreted?

Gould et al. (2022) and NASEM (2016) propose a model for using omics data in crop regulation with two possible outcomes decided by four categories of results from omics as summarized in Table 1. These categories are principally determined on the basis of the detection of differences in the omics profiles and the evaluation of these differences as potentially risky and interpretable.

As discussed above, the question of difference can be resolved experimentally so long as appropriate experimental standards are in place. However, the questions of whether differences are interpretable and whether they are likely to contribute to risk require more nuance. Interpretable and risky are somewhat subjective terms, and specific definitions may need to be decided for them through specific deliberative processes. How much uncertainty does there need to be in an interpretation of a difference for it to require further product testing? For example, Benevenuto et al. (2023) used comparative analyses and a systems biology approach for analyzing omics data of GE and non‐GE soybean lines. The GE variety was not equivalent to the non‐GE counterpart across statistical methods applied. They analyzed differentially expressed proteins for similarity to allergens, and 43 out of 78 differentially expressed proteins had at least 35% amino acid sequence identity to known allergens. This illustrates some gray areas between the categories in the NASEM (2016) report. Does 35% amino acid similarity to known allergens indicate enough of a potential risk to necessitate further testing (category 2 vs. category 3)? Or should these differences be considered uninterpretable because differences exist in multiple pathways and there is not necessarily specific knowledge of the potential ramifications of these pathways being altered (category 4)?

These categories provide a convenient framework, but drawing specific borders between categories involves decisions that are ‘more than technical’. As Kuzma (2016) reasons, “it is impossible to be completely ‘science‐based’ in a regulatory system,” and indeed, there is no purely scientific way of evaluating safety (Kuzma 2016). Distinctions that favor placing products in categories 1 and 2 represent a lower regulatory burden—as fewer products will undergo safety testing—but one that may limit the ability to generate risk hypotheses. Alternatively, distinctions that place more new products in categories 3 and 4 increase the regulatory burden. As more products undergo safety testing, the information from these tests will improve our ability to distinguish between categories as knowledge of how specific omics profiles correlate with safety improves.

## 3 Conclusion

Tiered omics regulatory systems have not begun to be implemented, and many of the specifics of these hypothetical systems have yet to be defined. Because of this, we illustrate a set of questions that are broadly applicable and illustrate that they are likely to impact various regulatory concerns. Among these impacts, we foresee a pattern where broader and deeper omics experimental requirements may benefit the scientific justification of a system and offer benefits for generating risk hypotheses but may also be more expensive and represent a higher regulatory burden. This tension may predicate deliberation about how these various regulatory concerns should be prioritized.

Taking a holistic view of these dilemmas opens yet another line of questioning—how will these decisions be made? And who will make them? These are of no less importance than the other questions, particularly when considering that many of the decision points highlighted here involve trade‐offs between competing regulatory concerns. In short, because the use of omics for public policy involves questions of values as much as technicalities, the processes and people by which decisions get made are of heightened importance (Holmes et al. 2010).

GE at large is one particular area of science and technology that has been routinely argued as a beneficiary of public engagement (Brossard et al. 2019; Burall 2018; Gould et al. 2022; Hurlbut et al. 2015; NASEM 2016; Parthasarathy 2015; Scheufele et al. 2021). In considering the use of omics technologies in the regulation of GE products, some may insist that the issue is so technically advanced that there is no practical benefit to inviting broader participation in its key decision points. To be sure, there are substantial practical challenges to engaging the public sphere, and if they are not carefully thought through, exercises can backfire in counterproductive ways (Kleinman et al. 2011). Nevertheless, there is a recent precedent for integrating interdisciplinary and cross‐sector perspectives into the formation and implementation of scientific experiments, which could offer insights into managing the complex set of questions we have introduced here (Kleinman and Suryanarayanan 2020).

Although the governance of GE crops remains politically divisive, gaining a more nuanced view of the choices and consequences can help guide further discussions, refinement, and evaluation of the tiered omics approach proposed by the NASEM committee. As an interdisciplinary group of researchers ourselves, we see great value in bringing together experts across different fields of study and institutional sectors, including developers, regulators, scientists, and consumers to move these discussions forward.

## Conflicts of Interest

The authors declare no conflicts of interest.

## Data Availability

The authors have nothing to report.
